# Factors Associated with Tuberculosis Treatment Default in an Endemic Area of the Brazilian Amazon: A Case Control-Study

**DOI:** 10.1371/journal.pone.0039134

**Published:** 2012-06-12

**Authors:** Marlucia da Silva Garrido, Maria Lucia Penna, Tomàs M. Perez-Porcuna, Alexandra Brito de Souza, Leni da Silva Marreiro, Bernardino Claudio Albuquerque, Flor Ernestina Martínez-Espinosa, Samira Bührer-Sékula

**Affiliations:** 1 Tropical Medicine Post-Graduate Program – Amazonas State University/Dr. Heitor Vieira Dourado Tropical Medicine Foundation, Manaus, Amazonas, Brazil; 2 Epidemiological Surveillance Department, Health Surveillance Foundation of Amazonas State, Manaus, Amazonas, Brazil; 3 Epidemiological Surveillance Department, Municipal Health Secretary of Manaus, Manaus, Amazonas, Brazil; 4 Epidemiology and Biostatistics Department, Universidade Federal Fluminense, Rio de Janeiro, Brazil; 5 Public Health Department, Medical School, Barcelona University, Barcelona, Catalonia, Spain; 6 Departament of Pediatrics-CAP Valdoreix, University Hospital of MútuaTerrassa, MútuaTerrassa Research Foundation, Universitat de Barcelona, Catalonia, Spain; 7 Epidemiology and Public Health Department/Dr. Heitor Vieira Dourado Tropical Medicine Foundation, Manaus, Amazonas, Brazil; 8 Leônidas and Maria Deane Institute, Oswaldo Cruz Foudation, Amazonas, Manaus, Amazonas, Brazil; 9 Tropical Pathology and Public Health Institute, Federal University of Goias, Goiania, Goias, Brazil; McGill University, Canada

## Abstract

**Setting:**

Treatment default is a serious problem in tuberculosis control because it implies persistence of infection source, increased mortality, increased relapse rates and facilitates the development of resistant strains.

**Objective:**

This study analyzed tuberculosis treatment default determinants in the Amazonas State to contribute in planning appropriate control interventions.

**Design:**

Observational study with a retrospective cohort using Brazilian Disease Notification System data from 2005 to 2010. A nested case control study design was used. Patients defaulting from treatment were considered as ‘cases’ and those completing treatment as ‘controls’. In the analysis, 11,312 tuberculosis patients were included, 1,584 cases and 9,728 controls.

**Results:**

Treatment default was observed to be associated to previous default (aOR 3.20; p<0.001), HIV positivity (aOR 1.62; p<0.001), alcoholism (aOR 1.51; p<0.001), low education level (aOR 1.35; p<0.001) and other co-morbidities (aOR 1.31; p = 0.05). Older patients (aOR 0.98; p = 0.001) and DOT (aOR 0,72; p<0.01) were considered as protective factor for default.

**Conclusions:**

Associated factors should be considered in addressing care and policy actions to tuberculosis control. Information on disease and treatment should be intensified and appropriate to the level of education of the population, in order to promote adherence to treatment and counter the spread of multidrug resistance to anti-TB drugs.

## Introduction

Tuberculosis (TB) is still a major public health problem. The reemergence of TB in the 1990s increased the need for innovative strategies for its control. Twenty-two countries account for 80% of cases of TB worldwide and Brazil is one of them with a prevalence rate of 38.1 cases per 100,000 inhabitants [1]. In Brazil, most patients are diagnosed in the state capital cities and metropolitan areas. The highest incidence rates are reported in the states of Rio de Janeiro and Amazonas, with 71.8 and 69.2 cases per 100,000 inhabitants, respectively [2].

The Amazonas State with an area of 1,570,745.680 km^2^ is located in Amazon region of Brazil and has a population of 3,483,985 inhabitants, with a density of 2.2 inhabitants per km^2^. The capital city, Manaus, harbors 51.7% of the population (1,802,014 inhabitants) with a population density of 158.06 inhabitants/km^2^ [3]. Sixty percent of the reported TB cases in the state reside in the capital. During the last decade, the cure and default rates of TB has been stable in the Amazon with averages of 66% and 11% respectively (State Program of Tuberculosis Control of the Amazon: unpublished data)

Treatment default is a serious problem in tuberculosis control. It may lead to persistence of infectious source, increased mortality, increased relapse rates, and facilitate the development of resistant strains [4], [5]. TB patients who are also illicit drugs users or alcohol abusers are more contagious and remain contagious longer because treatment failure presumably extends periods of infectiousness [6]. Mortality rates are high among TB patients who discontinue treatment, especially when associated with HIV infection [7]. The development of multidrug-resistant tuberculosis (MDR-TB), defined as resistance of the bacillus to at least isoniazid and rifampicin [1], is currently one of the biggest challenges for tuberculosis control.

To overcome this challenge, the World Health Organization recommends the adoption of the Directly Observed Treatment Short-Course strategy (DOTS) [8], in which one component is directly observed treatment (DOT) by a health professional. Several studies have reported that this measure reduces the abandonment of treatment [9]. However, other studies did not observe any difference between supervised or self-administered treatment [10].

Factors identified to be associated with treatment default are: lack of knowledge about the disease, distance from the health post, partial or complete regression of symptoms in the first two months of treatment, the side effects associated with the medication, male gender, age, the use of toxic substances and hospitalization during treatment [11]–[16], pre-existing pulmonary disease, previous default, TB/HIV co-infection [7], absence of supervised treatment and poor quality of patient care at the Health Unit [17], [18].

This study aimed to identify the associated factors linked to tuberculosis treatment default in the Amazonas State so as to contribute for a better planning intervention to control the disease.

## Methods

### Setting

**Figure 1:**
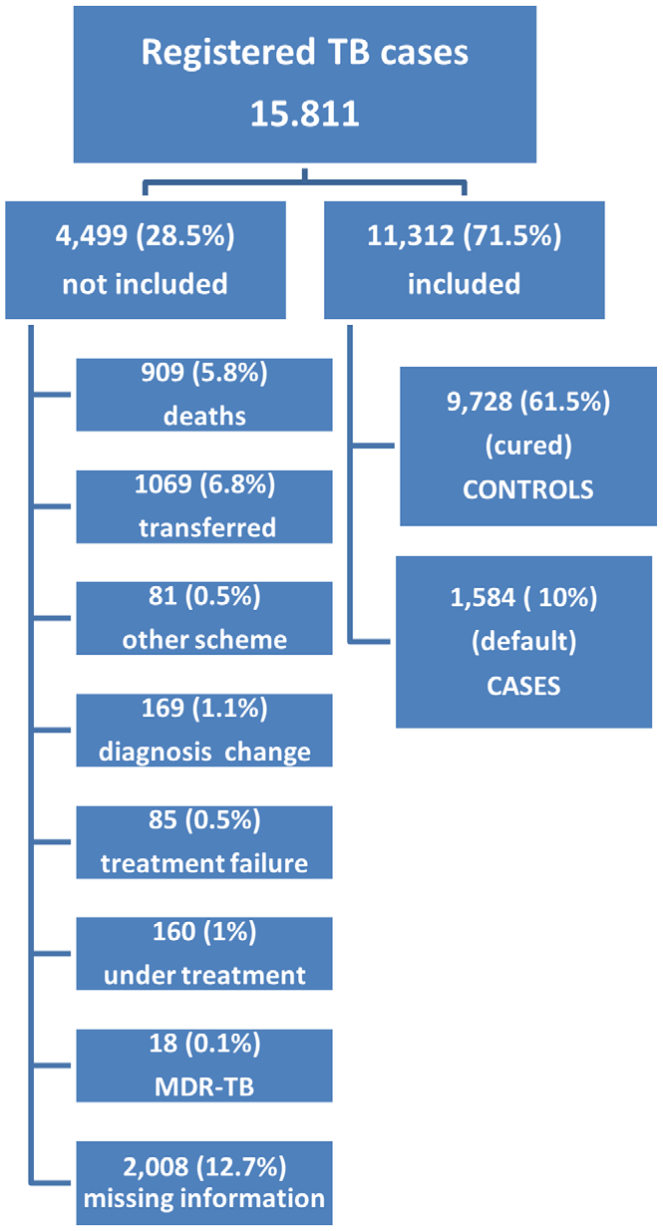
Registered TB patients and selection of cases and controls, Amazonas State, Brazil, 2005–2010.

This was an observational study with a retrospective cohort. Within the cohort a nested case control study design was used wherein patients defaulting from treatment were considered as ‘cases’ and those completing treatment as ‘controls’. Data from the National Notifiable Disease Information System (Sistema de Informação de Agravos de Notificação Compulsória) (SINAN) was used. All the children and the adults notified as tuberculosis cases from 2005 to 2010 in the Amazonas State were included in the study. The SINAN, created in 1993, is a national electronic surveillance system that contains a variety of diseases in an integrated database. This system accepts reports on cases and outbreaks and each case are reported individually. Relevant data are obtained from notifying health centers on standardized forms. Data are entered into the system and regularly updated, in most instances, by personnel from the Municipal Health Secretariats. These data are transferred electronically as described before by Penna et al. [19]. During the study period a total of 15,811 patients were notified at SINAN. The data comprehend the total number of cases diagnosed in the Amazon State during the study period. TB treatment is provided only by the Brazilian Government and it is free of charge drugs are not available in the pharmacies. The situation of treatment is recorded at 9^th^ month after the beginning of the treatment and it was considered for group selection. Therefore, 4,499 TB cases were excluded and 11,312 TB cases were selected (Figure 1). Of those 1,584 were defaulters and 9,728 completed their treatment.

### Definitions and statistical analysis

TB cases are notified according to definitions established by the Secretariat of Health Surveillance/Brazilian MoH and are based on recommendations of World Health Organization [20]. Defaulters were patients who interrupted treatment for two consecutive months or more, as defined by WHO and followed by the Brazilian MoH [20]. Patients are considered as cured when completing treatment according to Brazilian MoH standard protocols [20]. Active screening is recommended for HIV.

The variables studied were: age, sex, treatment performed in the living area, living in Manaus, living area, type of treatment, clinical form, sputum smear microscopy, HIV serology, Aids, alcoholism, diabetes, mental disorders, other co-morbidities, Direct Observed Treatment (DOT), education level, race.

The association of potential risk factors with defaulting was initially studied through univariate analysis. Tabulation generated univariate analysis and crude odds ratio (OR) was estimated as measure of association controlled by possible confounding variables. Variables statistically significant at 0.05 level were included in a logistic model to allow the estimation of adjusted OR for each variable controlled by others included in the model. Age was treated as a continuous variable and student “*t*” test was used to compare means in the univariate analysis. Data management and analysis was done using SPSS-version 10 and the statistical regressions were done using the *Statística* software [21] The sample of 1,573 cases and 9,438 controls has 80% of power to detect a relative risk of 1.2, alpha error of 5% and proportion of exposed among controls equals to 0.2.

### Ethical consideration

The study is a retrospective one using a secondary data bank from the online SINAN, prepared by the Brazilian Government with the purpose of epidemiological analysis in order to improve health care and disease control. Therefore, signed patients consent is not applicable. The study was approved by the Research Ethics Committee of the FMT-HVD, n° 2070 de 30/09/2011.

## Results

Table 1 shows the frequency of cases and controls according to year of enrollment. A total of 11,312 TB patients were enrolled in the study. Of these, 1584 were classified as cases and 9,728 as controls. There was no statistically significant difference in the number of cases enrolled between the years studied.

**Table 1:** Frequency of Tuberculosis Cases (defaulters) and Controls (cured) in the period of 2005 to 2010 – Amazonas State.

	Year of enrollment						
Situation	2005	2006	2007	2008	2009	2010*	TOTALS
Cases	305	266	287	288	260	178	1,584
	15%	13%	14%	14%	13%	15%	14%
Controls	1,724	1,763	1,755	1,766	1,721	999	9,728
	85%	87%	86%	86%	87%	85%	86%
TOTALS	2,026	2,028	2,040	2,053	1,980	1,176	11,312

2010* included cases that had information on treatment completion at SINAN in June 2011.

### Characteristics of study population

From the total study population 59.4% (6716/11312) are males and 69.4% (7854/11312) live in the capital Manaus. The mean age of the study population was 37.20 (SD 17.56). There were 89.5% (10032/11213) living in an urban area, 10.1% (1131/11213) in a rural area and 0.4% (45/11213) in a peri-urban area. Sputum smears were performed for 90.8% (10270/11312) of the TB patients. HIV serology was performed for 41.6% (4702/11312) and results were available for 34% (3842/11312) of patients. AIDS was reported in 5.7% (650/11312) of patients. DOT was used to 31.9% (2667/8362) of patients.

### Risk factors for default

Results of assessment on factors associated to default from TB treatment are shown in table 2.

**Table 2:** Univariate and Multivariate logistic regression analysis of factors independently associated with treatment default for tuberculosis patients from Amazonas State, Brazil 2005–2010.

		CASES	CONTROLS	Univariate analysis		Multivariate analysis	
				Crude OR (95% CI)		Adjusted OR (95% CI)	
		DEFAULTERS (%)	CURED (%)		p		p
Sex	Male	1059 (16.00)	5657 (84.00)	1.45(1.29–1.62)	0.00000	1.12(0.97–1.30)	0.11275
	Female	525 (11.00)	4071 (89.00)	1.00			
Treated living area	Yes	46 (14.00)	272 (86.00)	1.04			
	No	1538 (14.00)	9456 (86.00)	1.00	0.80946		
Living in Manaus	Yes	1195 (15.00)	6659 (85.00)				
	No	389 (11.00)	3069 (89.00)	1.42	0.00000	0.92(0.76–1.10)	0.35715
Living area	Urban	1426 (14.00)	8606 (86.00)	1.33			
	Rural	137 (12.00)	994 (88.00)	1.10			
	Peri-urban	5 (11.00)	40 (89.00)	1.00	0.13259		
Treatment type	Previous default	218 (47.00)	249 (53.00)	5.99	0.00000	3.20(2.25–4.57)	0.00000
	Relapse	61 (12.00)	427 (88.00)	0.98		0.68(0.43–1.08)	0.10140
	Transferred	71 (11.00)	604 (89.00)	0.80		0.74(0.48–1.13)	0.16792
	New case	1233 (13.00)	8440 (87.00)	1.00			
Clinical Form	Mixed	45 (18.00)	210 (82.00)	1.27	0.00014	1.21(0.82–1.78)	0.33584
	Extrapulmonary	163 (11.00)	1357 (89.00)	0.71		0.91(0.67–1.25)	0.56372
	Pulmonary	1376 (14.00)	8161 (86.00)	1.00			
AFB sputum smear	Negative	736 (15.00)	4158 (85.00)	1.17	0.02087	1.23(0.98–1.54)	0.07404
	Not done	140 (13.00)	902 (87.00)	1.02		0.90(0.67–1.21)	0.47094
	Positive	708 (13.00)	4668 (87.00)	1.00		1.00	
HIV	Positive	154 (21.00)	568 (79.00)	2.80	0.00000	1.62(1.38–1.89)	0.00000
	No results	174 (20.00)	686 (80.00)	2.62		1.47(0.67–2.19)	0.22405
	Not done	981 (15.00)	5629 (85.00)	1.80		1.05(0.32–1.91)	0.76982
	Negative	275 (09.00)	2845 (91.00)	1.00			
Aids	Yes	135 (21.00)	515 (79.00)	1.67(1.36–2.04)	0.00000	1.18(0.42–3.34)	0.50850
	No	1449 (14.00)	9213 (86.00)	1.00			
Alcoholism	Yes	196 (27.00)	527 (73.00)	2.56(2.13–3.07)	0.00000	1.51(1.25–1.84)	0.00003
	No	901 (13.00)	6197 (87.00)	1.00			
Diabetes	Yes	42 (10.00)	379 (90.00)	0.69(0.49–0.96)	0.02296	0.88(060–1.28)	0.49742
	No	1016 (14.00)	6297 (86.00)	1.00			
Mental disorders	Yes	24 (25.00)	71 (75.00)	2.17(1.32–3.59)	0.00088	1.15(0.61–2.14)	0.67120
	No	1026 (13.00)	6583 (87.00)	1.00			
Other comorbidities	Yes	104 (20.00)	422 (80.00)	1.58(1.26–1.99)	0.00005	1.31(1.01–1.71)	0.04547
	No	963 (13.00)	6188 (87.00)	1.00		1.00	
DOT	Yes	287 (11.00)	2380 (89.00)	0.66(0.57–0.76)	0.00000	0.72(0.55–0.94)	0.01437
	No	885 (16.00)	4810 (84.00)	1.00			
Education level	≤basic	1248 (15.00)	7019 (85.00)	1.43(1.26–1.63)	0.00000	1.35(1.15–1.57)	0.00017
	>basic	336 (11.00)	2709 (89.00)	1.00			
Race	Black	63 (16.00)	339 (84.00)	1.17	0.15334		
	Yellow	15 (18.00)	69 (82.00)	1.37			
	Mestizo	1209 (14.00)	7314 (86.00)	1.04			
	Indigenous	77 (11.00)	625 (89.00)	0.78			
	Ignored	15 (16.00)	78 (84.00)	1.22			
	White	200 (14.00)	1264 (86.00)	1.00			
Age	Continuous variable	37.32	36.45		0.71744	0.98(0.98–0.99)	0.00160

Treated living area: if the treatment was provided in the area where the patient lives. Living in Manaus: if the patient was living in the capital Manaus during treatment. Living area: the type of area where the patient lives: urban, periurban (near to urban areas but developing rural and urban activities), rural. Treatment type: the type of entrance of patient, if new case, if had received previous TB treatment but default, if was a relapse case or if transferred from another health unity. Clinical form: if TB case was pulmonary, extra pulmonary or mixed (pulmonary+extrapulmonary). AFB sputum smear: results of acid fast bacilli in sputum smear. HIV: results of serology or rapid test at moment of notification or during treatment. Aids: patients presenting Aids according WHO/MoH criteria at moment of notification. Alcoholism: condition recognized and declared by the patient at moment of notification. Diabetes (Diabetes mellitus): patient under treatment at moment of diagnosis, information registered in the patient file or declared by the patient. Mental disorders: condition declared at moment of diagnosis by patient's tutor or health professional or registered in the patient file. Other comorbidities: comorbidities not included in the standard questioner but declared by patients: heart disease, liver disease, kidney disease, neurological disease, cancer, mental disorders, and chemical dependency to illicit drugs. DOT: Directly Observed Treatment. Education level: ≤basic, comprehend illiterate and education up to 8 years of study; >basic: education above 8 years of study. Race: declared by patient at moment of notification. Age: Age of the patient at moment of diagnosis.

### Age and Gender

In univariate analysis males showed 45% greater risk of default, but this difference was not statistically significant in multivariate analysis. Multivariate analysis showed that the older the patients the lower the association with default, aOR 0.98; p = 0.001.

### Living and treatment area

It was not possible to show an association between treatment default and receiving treatment in a health unit located within the patient's living area or related to a patients location, an urban, peri-urban or rural area. Living in the capital Manaus seems to be associated with treatment default but it was statistically significant only in the univariate analysis.

### Clinical forms

Treatment default according to clinical form of the patient; default was observed in 18% of patients with mixed form (both pulmonary and extra pulmonary tuberculosis) of tuberculosis, 14% of patients with pulmonary tuberculosis and 11% of patients with extra pulmonary TB. In univariate analysis the difference was statistically significant, but lost significance in multivariate analysis.

### Sputum smear-microscopy

There was no association between sputum smear-microscopy and treatment default. Of those presenting negative sputum smear-microscopy 15% (736/4894) dropped out of treatment and 13% (708/5376) from those presenting positive microscopy.

### Conditions related factors

Co-infection with HIV/AIDS showed a statistically significant risk for default, aOR 1.62 (p<0.001). The risk of treatment default among people who report use of alcohol were higher than those who reported no alcohol use, aOR 1.51 (p<0.001). The chance of treatment default was 31% lower for diabetic patients, statistical significant only in univariate analysis, p = 0.02. In the univariate analysis patients with mental disorders and other co-morbidities are presented as risk factors, but only other co-morbidities remain in the multivariate analysis, aOR 1.31 (p = 0.04547).

### Type of treatment entrance

From patients reported as previous default, 47% (218/467) were recurring defaults. The risk of a previous case who has previously defaulted to default again was 3.2 times higher than the risk of a new case to default, (p = 0.000).

### Influence of learning opportunities

The probability to default was 28% lower for patients under DOT, aOR 0,72 (p = 0.01437). Treatment default risk was 35% higher for patients with lower education level, aOR 1.35 (p = 0.00017).

Race showed no influence on the risk for tuberculosis treatment defaulting.

## Discussion

Low cure rates and a high treatment default rate provide favorable conditions for the maintenance of disease transmission, high mortality and the development of resistant strains. These factors are observed in the Amazonas State, as well as an increase in the number of cases of primary and acquired multidrug resistance (Garrido et al, unpublished data).

Patients with TB in the Amazonas State are predominantly urban (88.7%), with the majority (69.4%) living in the capital Manaus. The large territory of the Amazonas State makes it difficult to control TB in riverside communities and indigenous areas, which rely on traveling hours to weeks by boat. The concentration of TB in the urban areas seems to favor disease control, probably due to its easy access to health services and treatment follow-up. However, this concentration of cases also favors the transmission of the disease. Therefore, understanding the factors leading to treatment default is of extreme importance to plan effective strategies.

Living in the capital (Manaus) appeared to be associated with noncompliance in univariate but in the multivariate analysis the association with treatment default was not confirmed. Jakubowiak at al. [22] observed in a case-control study of patients with smear-positive and negative groups that the risk of default by patients living in cities was not confirmed in the multivariate analysis. During the study period DOT's coverage in Manaus was lower than the coverage in the remote districts. Some of the remote districts have been historically performing supervised treatment for TB. Supervised treatment was implemented by the Special Public Health Service (PSAS) in partnership with the Institute of Interamerican Affairs in the decade of 40 (Decree Law no. 4275, 04/17/1942).

Our results were similar to those found by Jakubowiak et al [22] with respect to sex which was not identified as a risk factor for noncompliance in multivariate analysis. However, India and Kenya in the multivariate analysis showed sex as a risk factor [15], [23] for noncompliance. Our case-control studies have statistical power, and conflicting results may indicate differences in behavior among populations, which is beyond the scope of this analysis, but draws attention to the fact that the control actions must consider the particularities of each population. In the Amazon region, it is important to note the cultural differences, especially in indigenous areas.

In this study, we observed that the older patients were at lower risk of abandoning treatment. According to the model, each year of life reduced noncompliance by 2%. In a retreatment TB study including 2330 cases in India, Jha et al. found that patients aged 65 and above had a higher risk of dropping out treatment [23], unlike the results found by Jakubowiak et al in Russia, which points to higher risk for dropping out to be under 45 years of age [22]. In general, the studies showed that treatment default occurred mainly in the age group most affected by the disease, ie, in the economic productive age that may be linked to several factors such as family responsibility and enforcement of working hours.

There was no significant association between default and sputum smear-negative patient group (p = 0.07). TB/HIV coinfected patients that tend to develop paucibacillary TB often present negative smear results. Therefore, HIV infection may contribute to increase smear-negative cases but this association did not result in an association with smear negativity and treatment outcome in our analysis. AIDS did not remain as a risk factor after being inserted into the HIV positivity which appeared as a risk factor for default (aOR 1.62, p = 0.0000). These results are similar to those found in Kenya where TB/HIV co-infected patients had 56% higher risk for treatment default (aOR = 1.56, p = 0.0001) [15]. The TB/HIV drug combination can lead to treatment default as a result of the large number and diversity of drugs and adverse effects. The outcome of these cases can vary from deterioration of the clinical status to death. TB accelerates the evolution of HIV infection to AIDS by decreasing the patients' survival [24].

Alcoholism has been identified as an important predictor of treatment default in several continents. In India, the aOR was 1.72 (p = 0.002), in Russia, 1.99 (p = 0.04), in Africa, 4.97 (p = 0.01) and in Asia, 6.01 (95% CI 1.68 – 19∶47) [14], [15], [22], [25]. In our study, we found that people who reported use of alcohol had a 51% greater risk for treatment default (p<0.001) when compared to those who reported not using alcohol. The abuse of alcohol combined with antituberculosis drugs increases the risk of liver damage [26]. People involved with TB care, providers and communities, include associations that provide support to alcoholics and narcotics, psychological clinics and religious associations in the discussion of strategies to support patients which can help improve adherence to treatment as recommended by the WHO in the Stop TB strategy [8].

Diabetic patients had no increased risk of noncompliance. Univariate analysis showed a trend towards protection, which is confounded by the fact that diabetes is associated with older age [27] which also lowered the risk of treatment default. Other co-morbidities appear as a risk factor for treatment default (with OR 1.31, p = 0.04547) and the more frequent cited ones were smoking, alcoholism and illicit drug use. Oeltmann et al [6] point to the need for special attention to this group, they are more likely to be smear-positive combined with a higher risk of treatment failure, increasing the possibility of transmission. The probability of default is 3.2 times higher among patients who had already abandoned the previous treatment, p = 0.0000. Results of studies in India and Kenya were similar [22], [23]. Health professionals should consider that patients with prior noncompliance of treatment may not have received enough information about the disease and treatment. It is not hard to imagine that, in routine services with demand exceeding the capacity of practitioners, patients that have been already treated for tuberculosis do not receive adequate attention to strengthening the guidance on this disease and treatment. Mesfin et al. [18] showed that poor quality of TB service delivery and sub-optimal control district implementation of activities in public health facilities were key determinants of low adherence to treatment.

Muture et al found that inadequate knowledge was a significant factor for default. Receiving sufficient explanation on disease makes patients to understand treatment requirements, likely side effects to be encountered when using anti-TB drugs and the need to comply with treatment [15]. In our retrospective study this factor could not be addressed but the low education level and low DOT coverage suggests inadequate disease knowledge in our settings.

DOT coverage in the state of Amazonas in the period studied was 31.9% and the patients had overseen a 28% lower risk of treatment default than those who were not supervised, and this difference was statistically significant. However, we observed that even under DOT, 11% (287/2667) abandoned treatment. Moreover, the rate of 16% (885/5695) of dropout among cases that had self-administered treatment was similar to the 15.1% drop in group of patients under DOT observed by Mittal & Gupta [28] in India. The similarity of the results obtained with and without DOT, considering the difficulties of enforcement, raises issues related to quality and cost benefits of the strategy, which should be investigated in future studies designed for this purpose. Volmink J & Garner P point to the fact that no evidence exists to allocate resources as a routine supervised treatment until it is proven in situations where the benefit would be evident [10]. In Brazil, where the demand for health services does not allow full coverage of DOT, TB cases with positive microscopy results are prioritized.

Decentralization of tuberculosis control activities in Amazonas State started in the 90 s and was intensified in 2003 [29] in an attempt to improve care and treatment adherence. However, cure and abandonment rates remained stable. A good relationship between patient and health professional can improve treatment adherence, but there are several factors related to health care that may negatively affect adherence. They include negative attitudes of health professionals, lack of credibility and negative attitude of the patient in relation to services, lack of medicines and poor access to health services [17], [30]. The association of factors related to the health system with the studied predictors of abandonment should be considered when planning the control activities. The most significant factors associated with treatment default in this study were: poor education, the occurrence of default in the previous treatment, TB/HIV co-infection, alcoholism and other co-morbidities. Patients who have these conditions should be considered as targets for individualized attention and priority by health professionals, with emphasis on conducting DOT.

The study was not done with the intersection of mortality information system, so there may have been some cases of death which were incorrectly registered as abandoned. In addition, data was obtained from a public health database and some of the information might not be consistently captured. These may have introduced some bias and the results may have been a limitation of the study.

### Conclusions

The results presented as factors associated with abandonment: prior default, positive HIV serology, alcoholism, co-morbidities, failure to carry out DOT and low education level. These factors should be considered in addressing care and policy actions to tuberculosis control. Ensuring these patients receive DOT rather than expanding DOT to cover all groups may have more impact in our setting. Information about the disease and treatment are part of routine health services, but must be appropriate to the level of education of the population, in order to promote adherence to treatment and counter the spread of multidrug resistance to anti-TB drugs.
